# The impact of khat chewing on heart activity and rehabilitation therapy from khat addiction in healthy khat chewers

**DOI:** 10.1038/s41598-022-26714-w

**Published:** 2022-12-21

**Authors:** Ewunate Assaye Kassaw, Genet Tadesse Aboye, Daniel Yilma, Solomon Dhaba, Gizeaddis Lamesgin Simegn

**Affiliations:** 1 School of Biomedical Engineering, Jimma Institute of Technology Jimma University, Jimma, Ethiopia; 2 Internal Medicine Department, Jimma University Medical Centre, Jimma University, Jimma, Ethiopia; 3 Psychatry Department, Saint Paul’s Hospital Millennium Medical College, Addis Ababa, Ethiopia

**Keywords:** Biomedical engineering, Cardiovascular diseases, Cardiovascular diseases, Data processing, Image processing, Cardiology, Health care, Risk factors

## Abstract

Khat is a flowering plant whose leaves and stems are chewed for excitement purposes in most of east African and Arabian countries. Khat can cause mood changes, increased alertness, hyperactivity, anxiety, elevated blood pressure, and heart diseases. However, the effect of khat on the heart has not been studied exclusively. The purpose of this study was to investigate the impact of khat chewing on heart activity and rehabilitation therapy from khat addiction in healthy khat chewers. ECG signals were recorded from 50 subjects (25 chewers and 25 controls) before and after chewing session to investigate the effect of khat on heart activity. In addition, ECG signals from 5 subjects were recorded on the first and eightieth day of rehabilitation therapy for investigating the effect of rehabilitation from khat addiction. All the collected signals were annotated, denoised and features were extracted and analysed. After chewing khat, the average heart rate of the chewers was increased by 5.85%, with 3 subjects out of 25 were prone to tachycardia. 1.66% QRS duration and 23.56% R-peak amplitude reduction were observed after chewing session. Moreover, heart rate variability was reduced by 19.74% indicating the effect of khat on suppressing sympathetic and parasympathetic nerve actions. After rehabilitation therapy, the average heart rate was reduced by 11.66%, while heart rate variability (HRV), QRS duration, and RR interval were increased by 25%, 3.49%, and 12.53%, respectively. Statistical analysis results also confirmed that there is a significance change (*p* < 0.05) in ECG feature among pre- and post-chewing session. Our findings demonstrate that, khat chewing raises heart rate, lowers heart rate variability, or puts the heart under stress by lowering R-peak amplitude and QRS duration, which in turn increases the risk of premature ventricular contraction and arrhythmia. The results also show that rehabilitation therapy from khat addiction has a major impact on restoring cardiac activity to normal levels.

## Introduction

Khat (Catha edulis) is an evergreen flowering tree or shrub whose fresh leaves and delicate stems are chewed for recreational and stimulant purposes by populations in Ethiopia, Djibouti, Kenya, Somalia, South regions of Saudi Arabia and Yemen^1–3^. It is regularly consumed by about 20 million people^4–6^. In Yemen, for example, the habit of khat chewing is deep-rooted and involves at least 85% of the population, who consume khat on a daily basis lifelong. Chewing is also a habit among many youths in Ethiopia for its stimulation and euphoric effect^7^. Khat is listed as one of the drugs that create dependence (a containing desire to keep using it) in people by WHO^3, 7^. Due to this, it is a controlled substance in many countries including United states, Canada, Germany and the United Kingdom.

Even though, the environmental and climate conditions determine the chemical profile of khat leaves, many compounds are found in khat including alkaloids, terpenoids, flavonoids, sterols, glycosides, tannins, amino acids, vitamins and minerals^8^. It mainly contains chemicals called cathinone and cathine which produce stimulant effects^3^. Cathinone is mainly found in the young leaves and shoots. Cathinone and to less extent cathine are indirect sympathomimetic agents that trigger presynaptic dopamine release and reduce the reuptake of dopamine. Moreover, the substances elevate mood and produces euphoria^3, 7, 8^.

Khat is one of the drugs whose most common adverse effect is reported to occur on the cardiovascular system^9^. A research conducted in Yemen showed that khat chewing significantly increases the risk of acute myocardial infraction for heavy chewers up to 39-fold compared to non-chewers^10^. It was also reported that a an increase in diastolic and systolic blood pressure was observed in khat chewers^11^. Another study also found out that non-sustained ventricular tachycardia was observed on significant number of khat chewers^12^.

According to the research conducted in Ethiopia on selected 60 individuals with an average rate of chewing 1.7 times per week, 200 g of fresh “Beleche” khat showed a significant effect on inspiratory vital capacity (VCIN), forced vital capacity (FVC), forced vital capacity in one second (FVC1), the flow rate in the first one second (FEV1%), expiratory flow rate (FEF) and peak expiratory flow rate (PEFR)^13^. Moreover, the ventricular depolarization and conduction velocity was increased by 11%, the R-R interval reduced by 9%, and the QT interval reduced by 4.5%^13^. Likewise, according to a study conducted on 422 male chewers, it has been proved that, frequent chewers have an elevated systolic blood pressure possibility of 14 times more compared to less frequent chewers^14^. Khat chewers were more likely to have an elevated ST segment, higher risk of myocardial ischemia, cardiogenic shock, ventricular arrhythmia, and stroke compared with nonchewers^15^. Khat chewing was associated with the risk of an acute coronary syndrome, increased the risk of stroke and death^16^. In addition, for pregnant women chewing induces chest pain, tachycardia, and hypertension^17^.

Even though not all chewers are addicts, khat is a one of the addictive substances if it is excessively used. An addiction treatment is usually required to stop using khat. Rehabilitation therapy can help facilitate recovery from khat addiction and reduce the psychological and physiological disorder. The health conditions of 47 people rehabilitated from khat addiction in Saudi Arabia showed that quitting khat improved their health dramatically^18^. Despite the fact that the health service is always evolving in highly khat chewing countries, the rehabilitation therapy service for drug addiction appears to be under review.

In addition, even though literatures reveal the effect of khat on heart activity, the exclusive effect of khat on heart activity has not been conducted. The previous studies were conducted without considering the effect of caffeinated drinks taken while chewing khat and the effect of evaporation or losing constituents while extracting khat. For these reasons, it is difficult to conclude that the abnormal effects reported are exclusively due to khat use. Furthermore, the impact of rehabilitation therapy on khat addicts' heart activity has yet to be investigated. In this paper, the exclusive impact of khat on heart activity of healthy chewers has been investigated using a quasi-interventional design approach. In addition, the effect of rehabilitation therapy from khat addiction on heart activity has been investigated.

## Methods

**Figure 1:**
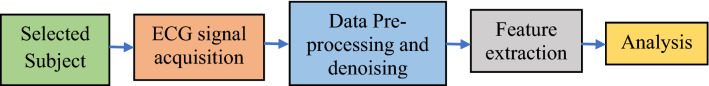
General procedures of the study.

To study the exclusive effect of khat chewing and rehabilitation therapy from khat addiction, ECG data was collected from healthy chewer subjects, control subjects and khat addicted subjects admitted to a rehabilitation centre. The variations in ECG signal features were extracted using signal processing techniques to analyse the changes in the cardiac activity. Figure 1 shows the general procedure used in this study.

### Study design

#### The effect of khat on heart activity

##### Sample selection and eligibility criteria

For the effect of khat on heart activity study, a quasi-interventional design approach was used. The subjects were selected based on a pilot survey conducted to identify appropriate subjects. The selection criteria and control procedures include (1) be able to comply to the study’s restrictions and conditions, such as not chewing khat and drinking alcohol for at least two days prior to the study, no coffee for the last 18 h, no soft drinks for the last 12 h, and no tea for the last 8 h, (2) being a young adult with ages between 18 and 35 (people in these age group are frequent chewers and also relatively healthy in Ethiopia), (3) should not have any confirmed case of cardiovascular diseases and is not taking any medications like lopinavir, ritonavir, azithromycin that could affect heart activity (a diagnosis was conducted prior to the study), and (4) free from any other drug use such as cocaine, marijuana, cannabis, and cigarette. A total of 50 subject (25 experimental and 25 control) were selected for this study. Among these, 38 of them were male, with 19 control and 19 chewer subjects; the remaining 12 were female, with 6 control and 6 chewer subjects. Their occupation includes students, health workers and civil servants.

##### Intervention characteristics

Chewer and control subjects were matched using criteria such as sex, age, body mass index (BMI), and occupation. To get the perfect match the control subjects were selected based on the chewers sex, age (± 5 years), BMI (less than 18.5 as underweight, 18.5–24.9 as normal, 25–29.9 as overweight and greater than 30 as obese). To reduce environmental factors that may alter heart activity, the ECG signals of matched chewer and control participants were recorded sequentially. Before ECG recording, all of the subjects were instructed to have their lunch. Each subject was instructed to take a 5-min rest to reduce the impact of any potential physical movements.

For the post-chewing session, initially khat leaves were prepared by removing any non-chewable components. Each chewer was given 100 g of the same type of khat called ‘Kellechaa’, which is the most widely available, preferred, and consumed khat in Jimma town. During the chewing session, tea, coffee, soft drinks, and cigarette smoking were prohibited. Both the intervention and control groups were exposed to similar interventional/care conditions. Post-chewing ECG data acquisition was conducted after 2 h of chewing session. The peak of excitement usually happens 2 h after the initial chewing session^19^. The chewers were also be able to pinpoint their highest excitation period.

##### ECG data acquisition

**Figure 2:**
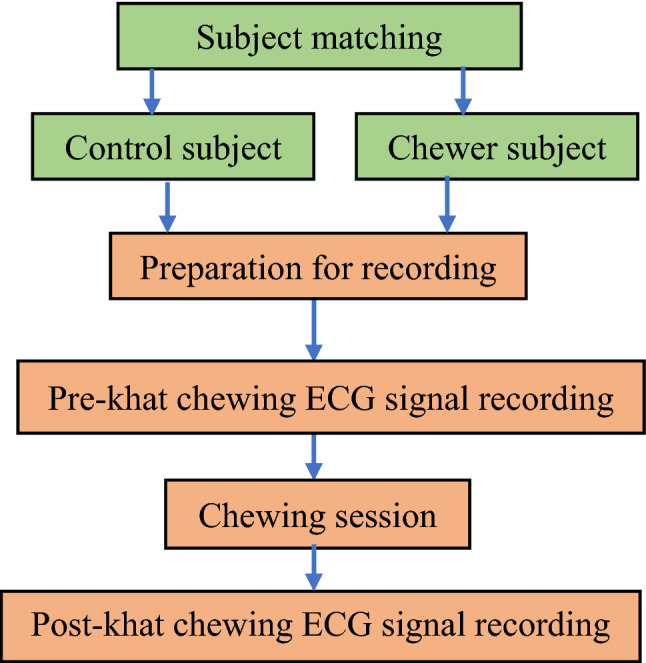
ECG data acquisition procedure to investigate the effect of khat chewing on heart activity.

Then, ECG data acquisition was conducted using Lead II while the participants are in a conventional sitting ECG recording position. Appropriate techniques and strategies were applied to improve signal quality, electrode skin interface conductivity, and reduce artefacts. Standard recording methods were used to setup the device and subjects for recording. Near the electrode placement areas, watches and jewellery were removed. Alcohol was used to clean the electrode attachment skin sites. The subjects were seated upright and relaxed prior to recording. The typical lead II configuration was used to place the leads. The negative electrode was placed on the side of the palm of the right forearm above the wrist, the positive electrode on the interior left leg just above the ankle, and the reference electrode on the interior right leg just above the ankle. Similar protocols were used to record ECG data for all sessions. A total of 100 ECG signals with 1-min duration were collected from 50 participants (25 control and 25 experimental). The ECG signal acquisition procedure for the khat chewing portion of the study is displayed in Fig. 2.

#### The effect of rehabilitation therapy from khat addiction on heart activity

For the rehabilitation therapy from khat addiction part of the study, data was obtained from khat addicts admitted to rehabilitation centres on the first day of admission and at the eighth day of admission for each subject. Most of the khat-addicted subject get recovered after a one-week stay in the rehabilitation program. In the rehabilitation centre, participants used to take medicines such as benzodiazepine, antidepressant or clonidine and therapies like watching TV, playing dart, playing table tennis, weight lifting, rope jumping and other sport activities as recommended by the physician. A case study approach was employed for this part of the study. The sample size was limited to 5 because most of the subjects with other co-addictions including alcohol, cigarettes, marijuana, opium, or a combination of one or more of these were excluded from the study. 10 ECG signals were recorded from these 5 subjects (1 female and 4 men), 5 before rehabilitation therapy and the remaining 5 after rehabilitation therapy.

### Ethical approval

The study was approved by institutional review board of Jimma institute of health, Jimma university, with permission number JHRPGN/75/21, and institutional review board of Saint Paul’s hospital millennium medical college, with approval number PM23/385. In addition, an informed written consent was obtained from all study participants prior to data collection. All methods were carried out in accordance with the ethical standards as laid down in the 1964 Declaration of Helsinki and its later amendments or comparable ethical standards.

### Study setting

For the effect of khat on heart activity part of the study, data collection for healthy control and chewer subjects was conducted in Jimma University Medical centre according to its intuitional protocol. For the effect of rehabilitation therapy from khat addiction on heart activity part of the study, data was collected in the psychiatric department of Saint Paul's Hospital Millennium Medical College and Addis Hiwot rehabilitation centre according to their respective protocols.

### ECG signal pre-processing

Following signal accusation, all the recorded ECG signals annotated and denoised. The recorded signals were given a unique name to identify the subject category, recording session, and counting number. Low-frequency noises caused by respiratory muscle movement, temperature change, and electrode motion artefact was removed. The Savitzky Golay filter was used to smooth the signal and remove low frequency disturbances^20^.

ECG signals are susceptible to disturbances such as powerline interference, EMG noise, and electromagnetic interference, in addition to baseline wandering abnormalities. As a result, any unwanted noises should be eliminated before extracting the relevant features. For our purpose, discrete wavelet transform which provides great performance for denoising ECG signals from these noises^21–23^ were used for signal denoising. Because the signals sampling frequency was 1000 Hz, the wavelet decomposition had frequency range patterns of 250–500 Hz, 125–251 Hz, 62.4–125 Hz, 31.2–62.6 Hz, 15.6–31.3 Hz, 7.79–15.7 Hz, 3.9–7.83 Hz, 1.95–3.92 Hz, 0.975–1.96 Hz, 0.487–0.979 Hz, 0.244–0.489 Hz, 0.0–0.243 Hz for detail (D) coefficients D1, D2, D3, D4, D5, D6, D7, D8, D9, D10, D11 and approximate (A) coefficient, respectively. Decomposition level 9 was used for eliminating frequency bands below 0.979 Hz that is assumed to be baseline wandering noise. The wavelet denoised signal was decomposed into wavelet coefficients and the energy of each coefficient was computed using a wavelet multiresolution analysis (MRA) technique to remove the residual noise. The low-frequency coefficients with a frequency range of less than 1 Hz which is out of the ECG signal range were excluded during wavelet reconstruction. Similarly, the frequency coefficients in ECG range having insignificant energy contribution were excluded during reconstruction. As a result, for reconstructing the denoised signal the contributor coefficients were selected from level 5 to level 9.

### Feature extraction

**Figure 3:**
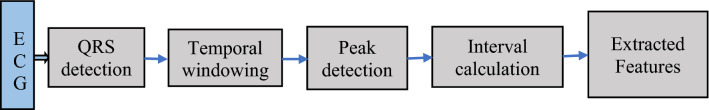
Feature Extraction model.

The temporal peak detection and interval calculation techniques were used to calculate the time domain ECG characteristics, following the Pan Tompkins QRS detection^24^ approach. Pan Tompkins algorithm is a time-domain QRS detection algorithm that consists of a series of lowpass filter, high pass filter, derivative filter, squaring, thresholding, and moving windowing procedures. The heart rate is calculated from the detected R peaks of the QRS complex. Figure 3 shows the feature extraction model employed in this study.

MATLAB peak detector functions “max” and “min” were used for detecting the location and amplitude of maximum and minimum peaks with in the calculated temporal moving windows. The maximum and the minimum amplitude points in each moving window were detected as R peaks and S peaks respectively. For Q wave the temporal window was between the left margin of the moving window and the R peak location, for P wave between the left margin of the moving window and the Q peak location and for T wave between S peak and right margin location of the moving window. The intervals and segments were calculated from the onset and offset points of each waves. The HRV was calculated using root mean square of successive differences (RMSSD) between each R-peak. Finally, all the important calculated features were exported to an excel format from MATLAB workspace for further analysis.

### Data analysis

The extracted features were averaged for better data manipulation. The changes between the averaged before and after chewing session for both chewers and controls were determined. Similarly, the changes between the averaged values before and after rehabilitation therapy were determined and the percentiles were computed from these values. In addition, the results have been statistically analysed using a pairwise t-test to show statistical differences among different groups (pre-chewing vs post-chewing of the experimental group, pre- vs post for the control group, pre-chewing of the experimental vs the control and post-chewing session of the experimental vs the control group).

### Informed consent

An informed written consent form was obtained from all study participants.

## Results

### Pre-processing

**Figure 4:**
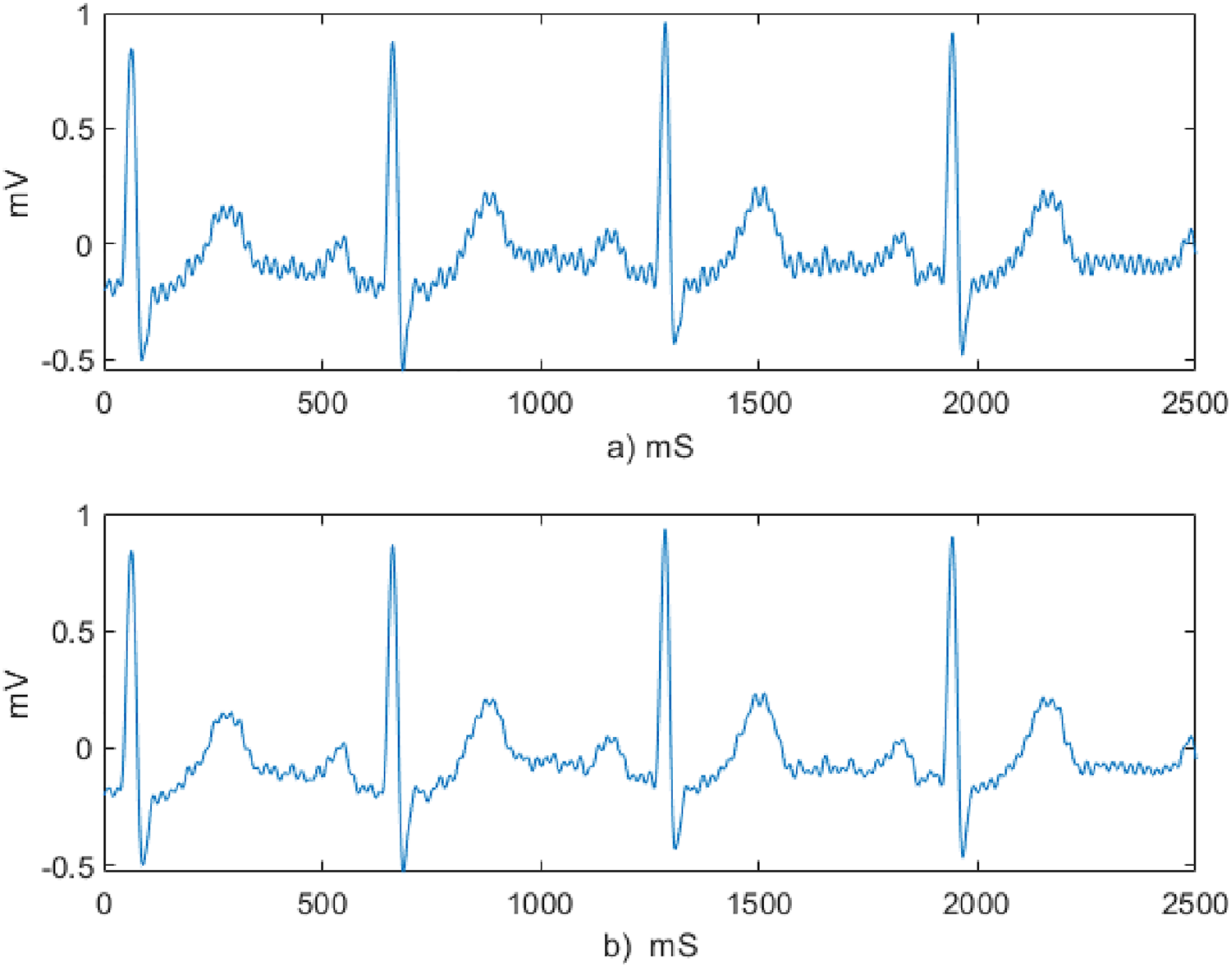
ECG signal denoising (**a**) sample raw ECG data of experimental group subject (**b**) the smoothed ECG signal using the Savitzky Golay filter.

The effect Savitzky Golay filter which was used to smooth the raw sample ECG data of one of the experimental group subjects is demonstrated in Fig. 4. High frequency noises which are observed in the raw ECG signal are reduced after filtering.

**Figure 5:**
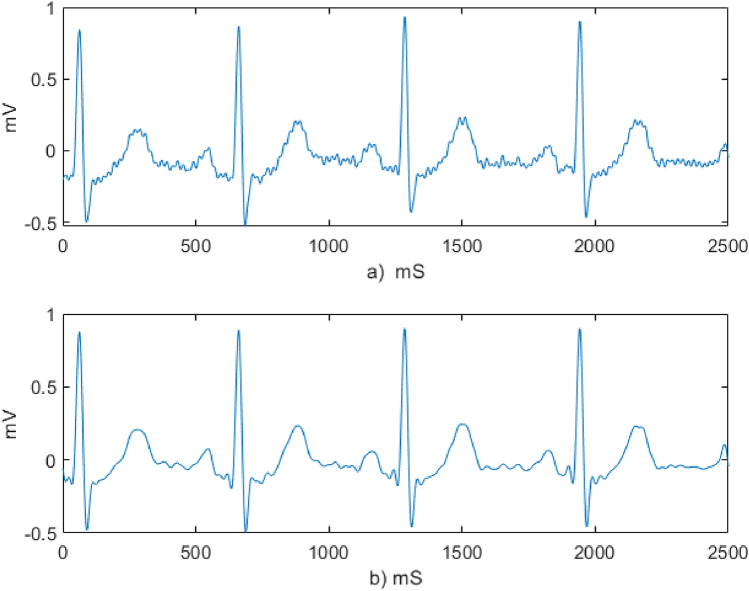
ECG signal denoising using Wavelet transform (**a**) input signal to wavelet multiresolution analysis (**b**) output signal of wavelet multiresolution analysis.

All the signals were denoised using discrete Meyer wavelet denoising technique followed by wavelet multiresolution analysis. Sample input and the output signals of the denoising process are demonstrated in Fig. 5.

### Feature extraction

**Figure 6:**
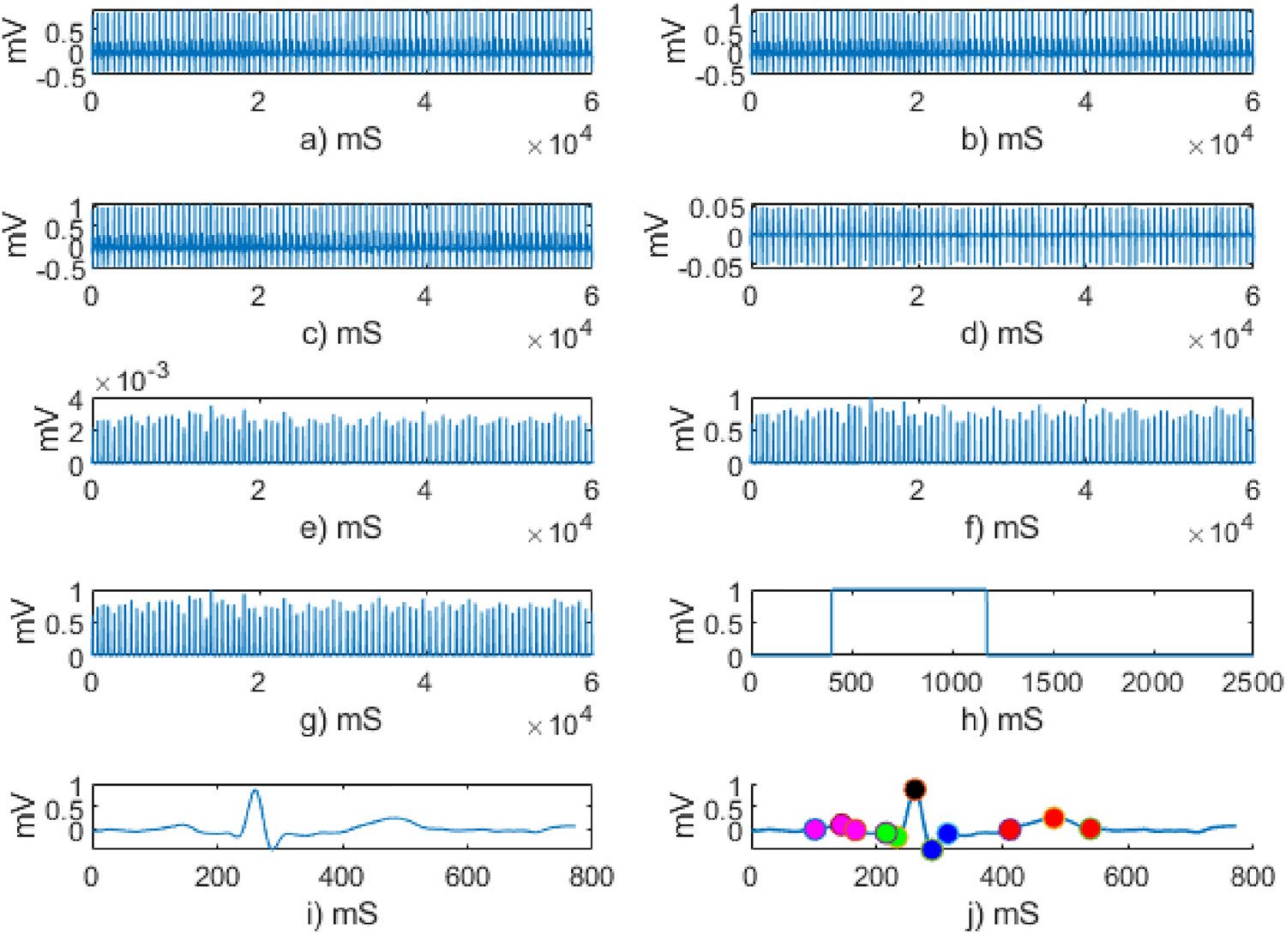
QRS detection using pan Tompkins: (**a**) Input signal, (**b**) low pass filtered (**c**) high pass filtered, (**d**) derivative filtered (**e**) signal squaring, (**f**) signal after normalization, (**g**) moving average filtered, (**h**) moving window and the portion of signal in the (**i**) moving window and (**j**) detected fiducial points.

By suppressing other signal components QRS peaks were successfully detected using the Pan Tompkins algorithm. After finding R peaks, temporal peak detection and interval calculation methods were used to determine the location and amplitude of other peaks. Figure 6 demonstrates the outcomes of various phases of the feature extraction process (QRS detection).

### Analysis results

#### The effect of khat on heart activity

Sample analysis results of the HRV values for the 25 experimental and control subjects, pre-chewing and post-chewing session are demonstrated in Table 1.

**Table 1:** Sample measurement results of HRV of the 25 experimental and control subjects, pre- and post-chewing sessions.

Subject (experimental/control)	HV of experimental		HRV of control	
	Pre-chewing	After chewing	Pre-chewing	Post-chewing session
1	0.00985628	0.007078574	0.011533333	0.009481228
2	0.007603504	0.003765549	0.005107496	0.006273889
3	0.007598881	0.005769079	0.004527778	0.008614346
4	0.003196429	0.005347421	0.007411394	0.007598881
5	0.005586667	0.003512805	0.007982443	0.007641764
6	0.007944183	0.004863581	0.006273889	0.005107496
7	0.006373565	0.00820676	0.00860676	0.00820676
8	0.001590769	0.001590769	0.008764706	0.006863014
9	0.009694444	0.006956013	0.008276036	0.011533333
10	0.004818568	0.004916912	0.0028	0.003450794
11	0.007411394	0.004925	0.008614346	0.004527778
12	0.003073276	0.004036839	0.007623044	0.007411394
13	0.002130618	0.003483343	0.007963633	0.007982443
14	0.003506667	0.003317683	0.003450794	0.002771014
15	0.007925373	0.003814286	0.007668298	0.00860676
16	0.009643836	0.00860676	0.009289855	0.013555556
17	0.003074074	0.003074074	0.009481228	0.008276036
18	0.00849277	0.008234099	0.002771014	0.0028
19	0.007553508	0.003468895	0.007641764	0.007963633
20	0.008873044	0.005447024	0.001557143	0.001585714
21	0.003184167	0.002975617	0.00820676	0.007668298
22	0.006907461	0.003671429	0.007598881	0.008873044
23	0.005756512	0.007668298	0.007	0.007
24	0.003301205	0.003301205	0.010633803	0.008797297
25	0.003246914	0.003246914	0.010141026	0.007445946

Table 2 illustrates the ECG feature average values for the chewer (experimental) and control subjects before and after chewing session. The second column presents the average features of the khat chewers ECG signals recorded before chewing session, the values of the third column are the average features of ECG signals recorded after chewing session, the fourth column is the difference of the after chewing session and before chewing session feature values and the fifth column presents the difference in percent. The sixth column presents controls average ECG signal feature values before chewing session, the seventh represents the controls ECG signal features recorded after the chewing session, the eighth shows the difference between after chewing and before chewing recorded ECG signals. The acronyms KB, KA, CB, and CA represent chewers before khat chewing, chewers after khat chewing, controls before khat chewing and controls after chat chewing session respectively. Table 3 demonstrates the statistical analysis (pair wise t-test) results of experimental and control subjects, pre-chewing and post-chewing sessions.

**Table 2:** Average ECG features of chewer and control subjects before and after chewing session.

Feature	Average KB	Average KA	KA-KB difference	% KA-KB difference	Average CB	Average CA	CA-CB difference	% CA-CB difference
Heart rate	78	81.76	3.76	4.82	71.24	71.52	0.28	0.39
HRV	0.005933	0.004851	0.001083	− 18.24	0.007237	0.007201	0.000036	− 0.49
R peak	0.837010	0.669003	0.168007	− 20.07	0.814931	0.833557	0.018626	2.29
QRS duration	0.090064	0.088810	0.001255	− 1.39	0.092046	0.091705	0.000341	− 0.37
P peak	0.084127	0.082114	0.002013	− 2.39	0.079387	0.079671	0.000285	0.36
T peak	0.164663	0.165019	0.000356	0.22	0.187729	0.195885	0.008156	4.34
PR interval	0.142974	0.135796	0.007178	− 5.02	0.150073	0.148826	0.001247	− 0.83
QT interval	0.315898	0.320796	0.004898	1.55	0.311215	0.311542	0.000327	0.11
RR interval	0.760514	0.731719	0.028795	− 3.79	0.829337	0.825766	0.003571	− 0.43
PR segment	0.058416	0.051427	0.006989	− 11.96	0.066368	0.065114	0.001253	− 1.89
ST segment	0.108749	0.106420	0.002328	− 2.14	0.101870	0.102531	0.000661	0.65

**Table 3:** Statistical analysis (*p* values) for experimental and control groups (pre-and post-chewing) for sample measurements.

Comparisons	HRV	Heart rate	QT interval	PR segment
Experimental pre-chewing versus post-chewing	0.006	0.01	0.002	0.0008
Control pre-chewing versus post-chewing	0.4	0.3	0.2	0.08
Experimental pre-chewing versus control pre-chewing	0.08	0.001	0.1	0.05
Experimental post-chewing versus control post-chewing	0.0009	0.0004	0.01	0.001

#### The effect of rehabilitation therapy from khat addiction on heart activity

Table 4 demonstrates the ECG feature average values of the 5 subjects before and after the rehabilitation therapy. The second and third columns represent the features ECG signal recorded before rehabilitation therapy and after rehabilitation therapy respectively. The acronyms RB and RA used for representing before and after rehabilitation therapy recorded ECG signals. the differences in number and percentage between before and after rehabilitation therapy are presented in columns four and five.

**Table 4:** The average values of ECG features before and after rehabilitation therapy.

Feature	Average RB	Average RA	RA-RB difference	%RA-RB difference
Heart rate	85.8	75.8	10	− 11.66
HRV	0.00564	0.007074	0.001434	25.43
R peak	0.765406	0.768712	0.003306	0.43
QRS duration	0.087332	0.090383	0.003052	3.49
P peak	0.064644	0.070426	0.005782	8.94
T peak	0.171017	0.166362	0.004655	− 2.72
PR interval	0.133617	0.138276	0.004659	3.49
QT interval	0.317281	0.317975	0.000694	0.22
RR interval	0.690836	0.777389	0.086553	12.53
PR segment	0.051404	0.056591	0.005187	10.09
ST segment	0.106831	0.115520	0.008689	8.13

Table 5 demonstrates the statistical analysis using a pair wise t-test results (*p* values) of experimental and control subjects, pre-chewing and post-chewing sessions.

**Table 5:** Statistical analysis (*p* values) between pre-rehabilitation and post-rehabilitation sample measurements.

Comparison	HRV	Heart rate	RR interval	PR segment
Before rehabilitation therapy versus After rehabilitation therapy	0.001	0.03	0.002	0.03

## Discussion

Khat is a plant whose leaves and stems are chewed in East African and Arabian countries, however it is outlawed in Europe and North America^3, 7, 8^. Acute myocardial infarction, increased blood pressure, raised ST segment, increased risk of myocardial ischemia, cardiogenic shock, ventricular arrhythmia, and stroke are all concerns associated with khat chewing^10, 11, 15, 16^. However, the exclusive effect of khat on cardiovascular disease has not been well studied in literature. In this study, ECG acquisition and processing techniques were employed to investigate the effect of Khat and rehabilitation therapy for khat addicted subjects on heart activity.

ECG signals were obtained from the chewer, control, and addicted subjects before and after chewing and rehabilitation therapy. After signal denoising, time domain feature extraction techniques were used to extract important ECG features including heart rate, heart rate variability, R peak, QRS duration, P peak, T peak, PR interval, QT interval, RR interval, PR segment, and ST-segment.

The results showed chewing khat increases heart rate by an average value of 5.85%. In addition, a decrement of 19.74%, 23.56%, 1.66%, 3.27%, 0.24%, 5.99%, 1.84%, 13.73% and 2.46% on heart rate variability, R peak amplitude, QRS duration, P-peak amplitude, T-peak amplitude, PR interval, QT interval, PR segment and ST-segment, respectively, were also observed. The statistical analysis result (Table 3) also demonstrates that the changes of HRV, heart rate, QT interval and PR segment were found to be more significant (*p* < 0.05) for the experimental group (pre- and post-chewing) compared to the control group. The results found are in agreement with a previous study that report an increment of heart rate and ventricular depolarization by 11% and decrement of RR interval by 9% due to khat chewing^13^. The reduction of heart rate variability is associated with lowered sympathetic and parasympathetic activities due to khat that caused cardiac stress^23, 25^. On the other hand, the reduction of R-peak amplitude and QRS duration is the sign of khat caused premature ventricular contraction or bundle branch blockage^13, 26^.

The heart rate and T-peak values were also shown to decrease after rehabilitation therapy, indicating an improvement on heart activity. One of the five participants was tachycardic with a heart rate of 99 beats per minute and restored to normal state (84 beats per minute) after rehabilitation therapy. The heart rate variability, R peak amplitude, QRS duration, P peak amplitude, PR interval, RR interval, PR segment, and ST-segment, showed considerable increment and restoration to the normal range after rehabilitation therapy. Moreover, the HRV, heart rate, RR interval and PR segment changes between pre-rehabilitation and post-rehabilitation measurements were all found to be significant (*p* < 0.05) as demonstrated in Table 5.

The results of this study shows that khat chewing has a significant negative effect on the electrical activity of the heart. The rehabilitation therapy from khat addiction indicates a positive effect on heart’s electrical activity. However, we acknowledge that our sample size is small and additional studies with a larger sample size are necessary. This study will help to aware community about the negative effects of khat as an input for the health care institutions and researchers. It was very challenging to get the positive response from the subjects for going to bioinstrumentation lab after chewing session. It is recommended to do this study by recruiting community representative subjects for better result.

## Conclusion

Khat is a flowering plant chewed for its stimulant and euphoric effects in most of eastern Africa and Arabian countries. Although it is widely viewed as a safe drug, it is linked to various cardiovascular disorders. The lack of proper laboratory studies regarding the impact of khat on heart activity and lack of studies on exclusive effect of khat make the interpretation of previous reports very difficult. In this study, ECG signal accusation and different processing techniques were employed to investigate the effect of khat chewing on cardiac activity. Different ECG features were extracted and used to determine the effect of chewing khat and rehabilitation from khat on heart activity. An average increment on heart rate and average reduction of heart rate variability, PR interval, RR intervals, PR segment, and ST-segment durations were observed after chewing khat. Moreover, heart rate variability was reduced by 19.74% indicating the effect of khat on suppressing sympathetic and parasympathetic nerve actions. Significant changes (*p* < 0.05) were also observed for most of the ECG feature measurements between pre-and post-intervention sessions for both investigations suggesting the significant effect of khat chewing on the electrical activity of the heart.

## Data Availability

The datasets used and/or analysed during the current study are available from the corresponding author on reasonable request.
